# Ethnic disparities in the association between maternal socioeconomic status and childhood anemia in Peru: a nationwide multiyear cross-sectional study

**DOI:** 10.1016/j.lana.2025.101117

**Published:** 2025-05-23

**Authors:** Ali Al-kassab-Córdova, Claudio Intimayta-Escalante, Pamela Robles-Valcarcel, Diego Urrunaga-Pastor, Baltica Cabieses

**Affiliations:** a Centro de Excelencia en Investigaciones Económicas y Sociales en Salud, Universidad San Ignacio de Loyola, Lima, Peru; b Facultad de Medicina de San Fernando, Universidad Nacional Mayor de San Marcos, Lima, Peru; c Facultad de Ciencias de la Salud, Universidad Peruana de Ciencias Aplicadas, Lima, Peru; d Universidad Científica del Sur, Lima, Peru; e Centro de Salud Global Intercultural, Universidad del Desarrollo, Santiago, Chile; f Department of Health Sciences, University of York, York, United Kingdom

**Keywords:** Indigenous, Mestizos, Afro-Peruvian, Socioeconomic status, Ethnic groups, Educational attainment, Inequalities, Peru

## Abstract

**Background:**

Maternal socioeconomic status (SES) is closely linked to children's health outcomes. However, the marginalization-related diminished returns theory suggests that increases in SES yield smaller health gains for marginalized populations—such as Afro-Peruvian and Indigenous groups—compared to majority groups like Mestizos, largely due to systemic barriers and social disadvantage. Therefore, the current study aimed to explore ethnic disparities in the association between maternal SES on childhood anemia in Peru.

**Methods:**

Using data from the 2017 to 2023 Peruvian Demographic and Health Survey, we conducted a cross-sectional study including children aged 6–59 months with their respective mothers. Ethnicity was grouped into Mestizo, Afro-Peruvian, and Indigenous (Quechua, Aimara, and native of the Amazon). Three proxies of SES were used: wealth index, level of education, and years of education. After stratifying by ethnicity, we estimated prevalence ratios (PR) with their respective 95% confidence intervals (95% CI) using generalized linear models with *Poisson* family. Interaction was assessed on multiplicative and additive scales.

**Findings:**

Among 234,364 Peruvian mothers, 45.7% (n = 107,118) identified as Mestizo, 12.6% (n = 29,557) as Afro-Peruvian, and 41.7% (n = 97,689) as Indigenous. The overall prevalence of anemia in children was 32.2%. The association between a very rich wealth index and lower prevalence of anemia was weaker among Indigenous (PR = 0.63, 95% CI: 0.56–0.72) compared to Mestizo individuals (PR = 0.46, 95% CI: 0.42–0.50). Similarly, the association between higher maternal education and lower anemia prevalence was less pronounced for Afro-Peruvian (PR = 0.70, 95% CI: 0.62–0.79) and Indigenous groups (PR = 0.81, 95% CI: 0.77–0.86) than for Mestizos (PR = 0.63, 95%CI: 0.59–0.67). A similar pattern was noted with maternal years of education (Mestizos [PR = 0.95, 95% CI: 0.94–0.96], Afro-Peruvian [PR = 0.97, 95% CI: 0.96–0.98], and Indigenous [PR = 0.98, 95% CI: 0.98–0.99]). Interaction analysis confirmed significantly weaker associations for Afro-Peruvian and Indigenous individuals compared to Mestizos.

**Interpretation:**

Maternal SES is associated with lower prevalence of childhood anemia, with stronger associations observed among Mestizo populations compared to Afro-Peruvian and Indigenous groups. This pattern aligns with the marginalization-related diminished returns theory. Maximizing SES alone does not preclude ethnic disparities but rather, may even widen them, highlighting the need for equity-focused interventions that address underlying structural and systemic barriers.

**Funding:**

Self-funded.

**Research in context**

**Evidence before this study**

About two-thirds of Peruvian children suffer from anemia, with prevalence varying across ethnic groups. As a multiethnic country, Peru faces significant socioeconomic disparities that disproportionately impact Afro-Peruvian and Indigenous populations compared to Mestizos. The marginalization-related diminished returns phenomenon posits that ethnically disadvantaged individuals derive fewer benefits from the same socioeconomic resources, such as education and wealth, due to ongoing societal and structural barriers.

We searched PubMed for studies on the marginalization-related diminished returns phenomenon in Latin America, with no restrictions, for publications before February 2025. The search strategy used was: ((“diminished returns”) AND (“Latin America”)). Although we found no published studies on this topic, extensive evidence reported in the United States suggests that this phenomenon is also likely occurring in Latin American countries such as Peru.

**Added value of this study**

This population-based study examined the protective role of maternal socioeconomic status on anemia among Peruvian children from the country's three largest ethnic groups: Mestizo, Indigenous, and Afro-Peruvian. The protective effect was weaker for Afro-Peruvian and Indigenous, as compared to Mestizo individuals. In Peru, *diminished returns* stem from *differential exposures* and *differential effects*, including disparities in education quality and access, marginalization, economic exclusion, discrimination, and violence.

**Implications of all the available evidence**

Simply increasing maternal education and wealth may unintentionally widen ethnic disparities in childhood anemia, as the benefits of these socioeconomic resources are hindered by structural barriers affecting Indigenous and Afro-Peruvian populations. Equity-focused policies at both economic and social levels should include interventions that address underlying structural and systemic barriers. The *diminished returns* phenomenon should be further investigated in other Latin American countries.

## Introduction

Despite decades of progress, anemia continues to cast a long shadow over childhood health. Although the global prevalence of anemia decreased from 28.2% in 1990 to 24.3% in 2021, it still affects 41.4% of children under five worldwide.^1^ Iron deficiency, mainly produced by insufficient intake of iron-rich foods, is the primary cause of anemia in children, especially in low- and middle-income countries.^2^^,^^3^ This condition, which disproportionately affects children living in poverty, hinders psychomotor, and cognitive development, with long-term consequences for educational performance, professional growth, and quality of life.^4^, ^5^, ^6^, ^7^ These long-term effects further reinforce social and economic inequalities. In Peru, childhood anemia remains a critical issue, with one of the highest prevalences among children under 5 years of age in Latin America. These rates vary according to maternal characteristics such as the region of origin, educational level, socioeconomic status (SES), and ethnicity.^8^, ^9^, ^10^ Previous studies have described socioeconomic disparities in the prevalence of anemia among children under 5 years old in Peru, which are even more pronounced among different ethnic groups.^11^^,^^12^ This situation may be due to structural barriers.^13^

According to the marginalization-related diminished returns theory, proposed by Assari,^14^ structural barriers prevent ethnic minorities from fully benefiting from higher SES, limiting its protective effects on health.^14^ Even with advances in maternal SES, such as increased maternal education or household income, Afro-Peruvian and Indigenous individuals may see fewer health benefits than their Mestizo counterparts because of discrimination, social stratification, segregation, economic constraints, geographic isolation, limited access to quality resources, and inadequate health infrastructure.^15^^,^^16^ In other words, these barriers would prevent mothers from disadvantaged ethnic groups to translate socioeconomic gains into better child health outcomes, increasing the risk of childhood anemia. For example, Peruvian Afro-descendants have lower access to improved water and sanitation, institutional delivery, early prenatal care, and birth registration. They also experience higher rates of adolescent maternity and stunting compared to their non-Afro-descendant counterparts.^15^ Similarly, Indigenous populations face these challenges but also tend to reside in remote rural areas, further limiting their access to healthcare. Additionally, they are far more likely to avoid healthcare services due to experiences of mistreatment, not only due to language barriers but also related to discrimination based on their rural background, socioeconomic status, attire, and manner of speech.^17^^,^^18^ As a consequence, the persistence of these inequalities hampers the progress of government initiatives, such as micronutrient supplementation and improved access to healthcare, to tackle anemia.^19^^,^^20^

Afro-Peruvian and Indigenous communities are among the most historically marginalized and socioeconomically disadvantaged ethnic groups in Peru. Afro-Peruvians have endured the lasting impacts of slavery, which subjected them to generations of exploitation and dehumanization. Even after abolition, systemic racism, and social exclusion has persisted.^21^ The Indigenous population of Peru—including Quechua, Aimara, and natives of the Amazon—have also historically faced inequities rooted in colonization.^22^ These processes established structural domination, marginalization, and wealth extraction, which have transcended generations, significantly limiting access to healthcare, education, and essential resources.^13^^,^^15^^,^^23^ These conditions exacerbate health inequities, increasing the risk of anemia and underscoring the need for targeted interventions in vulnerable ethnic groups. Therefore, we examined whether the association between maternal SES and childhood anemia varies among Afro-Peruvian, Indigenous, and Mestizo Peruvian children, through the lens of the marginalization-related diminished returns framework, using a nationally representative dataset.

## Methods

### Study design

We performed a cross-sectional analytical study using data from the 2017 to 2023 Peruvian Demographic and Health Surveys (DHS). The DHS is a probabilistic survey conducted annually by the National Institute of Statistics and Informatics (INEI, from the Spanish acronym). The DHS collects sociodemographic and health information on mothers and children. The sampling design is stratified, two-stage, and independent. The level of representativeness is national, regional, and by area of residence (urban and rural). The data were collected through direct interviews, except for the 2020 DHS, in which participants were contacted by phone due to the restrictions of the COVID-19 pandemic. Biomarker collection, including hemoglobin testing, was conducted in person once mobility restrictions were eased in 2020. The reporting of this study is aligned with the STROBE (Strengthening the Reporting of Observational Studies in Epidemiology) guidelines (see Suppl. Table S1). This study is based on secondary analysis of publicly available, de-identified data from the Peruvian DHS. Given the anonymized and public nature of the data, ethical approval was not required.

### Participants

The study population consisted of Peruvian child–mother pairs included in the 2017–2023 DHS surveys. In the DHS, children were eligible for inclusion if they were under five years old (0–59 months), were usual residents of the selected household or had stayed there the previous night, and had parental or guardian consent for participation, including biomarker collection such as hemoglobin testing. For our study, we included child–mother pairs if the child was 6–59 months old and both the child and mother had complete data for all the variables of interest (complete-case analysis). We excluded a child–mother pair if the mother self-identified as “White”, “Other”, or “Do not know”, as these groups had small sample sizes and did not align with our research objective.

### Setting

Peru is an upper-middle income country with 34 million inhabitants located in South America.^24^ Administratively, it is divided into 24 regions and one constitutional province and further subdivided into provinces and districts. Geographically, the country is divided into the Coast (bordering the Pacific Ocean), the Highlands (dominated by the Andes Mountains), and the Jungle (divided into high and low jungle). One-third of the population is concentrated in Lima, the capital of Peru.

Peru is a multiethnic nation characterized by a rich tapestry of cultural identities. According to the 2017 National Census, 60.2% identify as Mestizo, 25.3% as Indigenous (22.3% Quechua, 2.4% Aimara, and 0.6% natives of the Amazon), 3.6% as Afro-Peruvians, and the remaining population identifies with other ethnic groups.^25^ Mestizos, individuals of mixed Indigenous and European (mainly Spanish) descent, predominantly reside in urban coastal areas and speak Spanish. Afro-Peruvians, descendants of African slaves brought to Peru during the colonial period, also speak Spanish and are based in urban coastal areas.^21^ Regarding Indigenous individuals, they often speak their native languages or dialects, maintain traditional health beliefs, and are primarily based in rural areas of the Andean highlands and the Amazonian jungle. The Quechua, descendants of the Inca civilization, are the largest Indigenous group in Peru, with about five million people. The Aimara, a pre-Inca civilization, reside in the high plateau region of the southern Peruvian Andes and extend into Bolivia and Chile. Amazonian Indigenous groups, including the Shipibo-Conibo, Asháninka, and Aguaruna, have a deep cultural and spiritual bond with the rainforest, now increasingly at risk.^22^^,^^25^ All Indigenous groups experience similar social, economic, and political barriers; therefore, they were grouped in the main analyses.

### Outcome

Anemia was defined according to the guidelines of the World Health Organization (hemoglobin level less than 11 g/dL).^26^ The same definition is used by the Peruvian Ministry of Health. Hemoglobin levels were measured with a HemoCue® Hb201 portable hemoglobinometer, and subsequently adjusted for altitude using the formula provided by the US Centers for Disease Control and Prevention.^27^

### Socioeconomic status

Three indirect measures of maternal SES were considered: level of education, years of education, and wealth index.^28^ While correlated, these variables require differential interpretation due to different causal mechanisms.

• Wealth index: This is a composite measure of the cumulative living standard of a household and is calculated using data on household assets, utilities and housing features. After gathering these data, principal component analysis is used to create the wealth index. This method transforms these variables into a set of scores and each household is given a score. Households are then ranked according to their wealth scores and divided into 5 quintiles: “poorest”, “poorer”, “middle”, “rich”, and “richest”. The wealth quintiles are defined for the entire population, which is preferable; however, it should be clarified that while each quintile represents 20% of households, here we present the distribution based on children rather than households. Since poorer households tend to have more children, the distribution skews accordingly.
• Level of education: The maximum level of education attained was classified into “none or primary”, “secondary”, and “higher”. In Peru, education is freely provided by the Ministry of Education. Primary education in Peru comprises 6 years, secondary education consists of 5 years, and higher education can either be technical, lasting 3 years, or university-level, typically spanning 5–7 years.
• Years of education: Numbers of years of formal education, ranging from 0 to 18.

### Covariates

Using Direct Acyclic Graphs, we selected potential confounders—common causes of SES proxies and anemia—considering our empirical assumptions, knowledge and the current literature. Thus, we included the age of the child and mother (in months and years, respectively), sex (male/female), area of residence (urban/rural), region of residence (Lima, rest of Coast, Highland, Jungle), family size (≤4/>4 members), disability (yes/no), partner status (with partner/without partner), employment status (currently working, working last year, not working), and health insurance (yes/no) (see Suppl. Figure S1).

### Ethnicity

The Peruvian DHS collects information on ethnicity by asking this question: “Due to your customs and your ancestors, do you feel or consider yourself … ?”. The potential answers were: “Quechua”, “Aimara”, “Native of the Amazon”, “part of another Indigenous or native people”, “Afro-Peruvian”, “White”, “Mestizo”, “Other”, and “do not know”. To fit the analyses, we categorized autochthonous ethnical groups (Quechua, Aimara, and natives of the Amazon) as Indigenous. Only Afro-Peruvian, Indigenous, and Mestizos were included in this study.

### Statistical analysis

We performed the analysis in STATA version 18 considering the complex survey design. This approach properly incorporates sampling weights, stratification, and clustering, ensuring that estimates are representative of the national population and that standard errors are correctly adjusted for intra-cluster correlations.

To describe and summarize the data, we calculated weighted proportions for categorical variables and weighted means with standard deviations (SD) for continuous variables. To assess the association between maternal SES and childhood anemia, we estimated adjusted prevalence ratios (PR) with their respective 95% confidence intervals (95% CI) using generalized linear models with *Poisson* family and robust variance estimation. The reference category for each covariate was chosen based on theoretical relevance and prior literature. It was typically the lowest risk or most disadvantaged category to facilitate meaningful comparisons. The PR is a measure of association that represents the relative difference in the probability (prevalence) of anemia between exposure groups. A PR greater than 1 indicates a higher prevalence of anemia in the exposed group compared to the reference category, while a PR less than 1 suggests a lower prevalence.^29^ Additionally, we calculated the predicted prevalence of childhood anemia, based on the fitted model. This approach provides an adjusted estimate of the prevalence while holding the other variables at their observed values. The analyses were stratified by ethnicity, SES category and survey year.

To examine whether the association between maternal SES and childhood anemia varies across ethnic groups, we incorporated interaction terms between ethnicity and SES indicators in separate models with different interaction terms of *SES* × *ethnicity*, with Mestizo children serving as the reference group. We assessed interaction on both the multiplicative and additive scales, as recommended by Know & VanderWeele.^30^ On the multiplicative scale, we reported PR with 95% CI, while on the additive scale, we calculated the relative excess prevalence due to interaction (RERI). RERI quantifies the excess prevalence resulting from the joint effect of SES and ethnicity, beyond the sum of their individual effects. It is calculated using the following formula: $RERI={\mathrm{PR}}_{SESxethnicity}-\left({\mathrm{PR}}_{SES}+{\mathrm{PR}}_{ethnicity}-1\right)$. In this formula, ${\mathrm{PR}}_{SESxethnicity}$ represents the PR for the combined exposure (e.g., high wealth index and Afro-Peruvian ethnicity), ${\mathrm{PR}}_{SES}$ is the PR for SES alone (e.g., high SES regardless of ethnicity), and ${\mathrm{PR}}_{ethnicity}$ is the PR for ethnicity alone (e.g., being Afro-Peruvian regardless of SES). The value of the RERI indicates the nature of the additive interaction: a RERI greater than zero suggests a positive interaction (more prevalence than expected), a RERI of zero indicates no interaction, and a RERI less than zero indicates a negative interaction (less prevalence than expected).^31^

### Role of funding source

There was no funding source for this study.

## Results

### Description of participants

We included data from 234,364 Peruvian mothers, of whom 45.7% self-identified as Mestizo (n = 107,118), 12.6% as Afro-Peruvian (n = 29,557), and 41.7% as Indigenous (n = 97,689) (see Suppl. Figure S2). The average age of the mothers was 33.1 years (SD: 6.9), whereas the children had an average age of 34.1 months (SD: 15.7), with 51.1% (n = 119,611) of the children being male. Regarding the education levels of mothers, 45.6% (n = 106,908) had attained a secondary education. The prevalence of childhood anemia was 32.2% (n = 81,026), being higher in Afro-Peruvian (30.3%, n = 9336) and Indigenous (39%, n = 39,193), in comparison to the Mestizo children (28.3%, n = 32,497). The characteristics of the participants substantially differed according to ethnicity (see Table 1). In addition, the baseline characteristics of the different Indigenous groups are described in Suppl. Table S2.

**Table 1:** Characteristics of the participants, 2017–2023 DHSs.

Characteristics	All Peruvian mothers with children included (n = 234,364)	Mestizo (n = 107,118)	Afro-Peruvian (n = 29,557)	Indigenous (n = 97,689)
	n (%)	n (%)	n (%)	n (%)
**Mother's age (mean [SD]**)	33.1 [6.9]	33 [6.7]	32.6 [7.1]	33.5 [6.9]
**Level of education**				
None or primary	65,625 (25.5)	17,880 (15.4)	11,411 (40.6)	36,334 (34.4)
Secondary	106,908 (45.6)	49,903 (46.3)	13,718 (44.5)	43,287 (44.9)
Higher	61,831 (28.9)	39,335 (38.3)	4428 (14.9)	18,068 (20.8)
**Wealth index**				
Poorest	78,451 (28.1)	21,429 (17.4)	11,328 (38.6)	45,694 (39.7)
Poor	62,516 (24)	27,708 (21.5)	8819 (27.2)	25,989 (26.5)
Middle	43,571 (19.4)	23,512 (21.2)	5320 (17.6)	14,739 (17.4)
Rich	30,461 (16.1)	19,909 (21.2)	2868 (11.3)	7684 (10.5)
Richest	19,365 (12.4)	14,560 (18.7)	1222 (5.3)	3583 (5.8)
**Area of residence**				
Urban	155,579 (71.6)	86,276 (83)	18,969 (63.4)	50,334 (57.9)
Rural	78,785 (28.4)	20,842 (17)	10,588 (36.6)	47,355 (42.1)
**Region of residence**				
Lima	25,218 (26.1)	17,139 (34.6)	2284 (14.5)	5795 (18.2)
Rest of the coast	63,590 (25.2)	38,148 (30.2)	13,405 (44.7)	12,037 (9.3)
Highland	81,681 (28.6)	15,404 (12)	6691 (23.5)	59,586 (55.9)
Jungle	63,875 (20.1)	36,427 (23.2)	7177 (17.4)	20,271 (16.5)
**Physical or mental disability**				
None	233,109 (99.4)	106,562 (99.4)	29,441 (99.6)	97,106 (99.4)
At least one	1255 (0.6)	556 (0.6)	116 (0.4)	583 (0.6)
**Partner status**				
No	32,789 (14.1)	16,794 (15.4)	4363 (14.6)	11,632 (11.9)
Yes	201,575 (85.9)	90,324 (84.6)	25,194 (85.4)	86,057 (88.1)
**Employment status**				
Not working	69,707 (31.4)	33,881 (32.6)	10,955 (38.8)	24,871 (26.4)
Working	19,575 (8.8)	9469 (9.2)	2764 (9.8)	7342 (7.9)
Work in last year	137,507 (59.8)	60,461 (58.2)	15,021 (51.4)	62,025 (65.7)
**Family size (members)**				
≤4	85,706 (35.6)	39,150 (35.8)	9937 (32.2)	36,619 (36.9)
>4	148,658 (64.4)	67,968 (64.2)	19,620 (67.8)	61,070 (63.1)
**Health insurance**				
No	28,970 (14.2)	15,538 (15.8)	3339 (12.9)	10,093 (12.4)
Yes	205,394 (85.8)	91,580 (84.2)	26,218 (87.1)	87,596 (87.6)
**Sex of children**				
Male	119,611 (51.1)	54,736 (51)	14,998 (51.2)	49,877 (51.2)
Female	114,753 (48.9)	52,382 (49)	14,559 (48.8)	47,812 (48.8)
**Child's age (mean [SD])**	34.1 [15.7]	34.1 [15.6]	34.3 [15.6]	34.2 [15.5]
**Anemia**				
No	153,338 (67.8)	74,621 (71.7)	20,221 (69.7)	58,496 (61)
Yes	81,026 (32.2)	32,497 (28.3)	9336 (30.3)	39,193 (39)

SD: standard deviation.

**Note:** Proportions and means are weighed to account for the complex survey design. Mother's age is reported in years; child's age is reported in months.

### Association between wealth index and childhood anemia

**Fig. 1:**
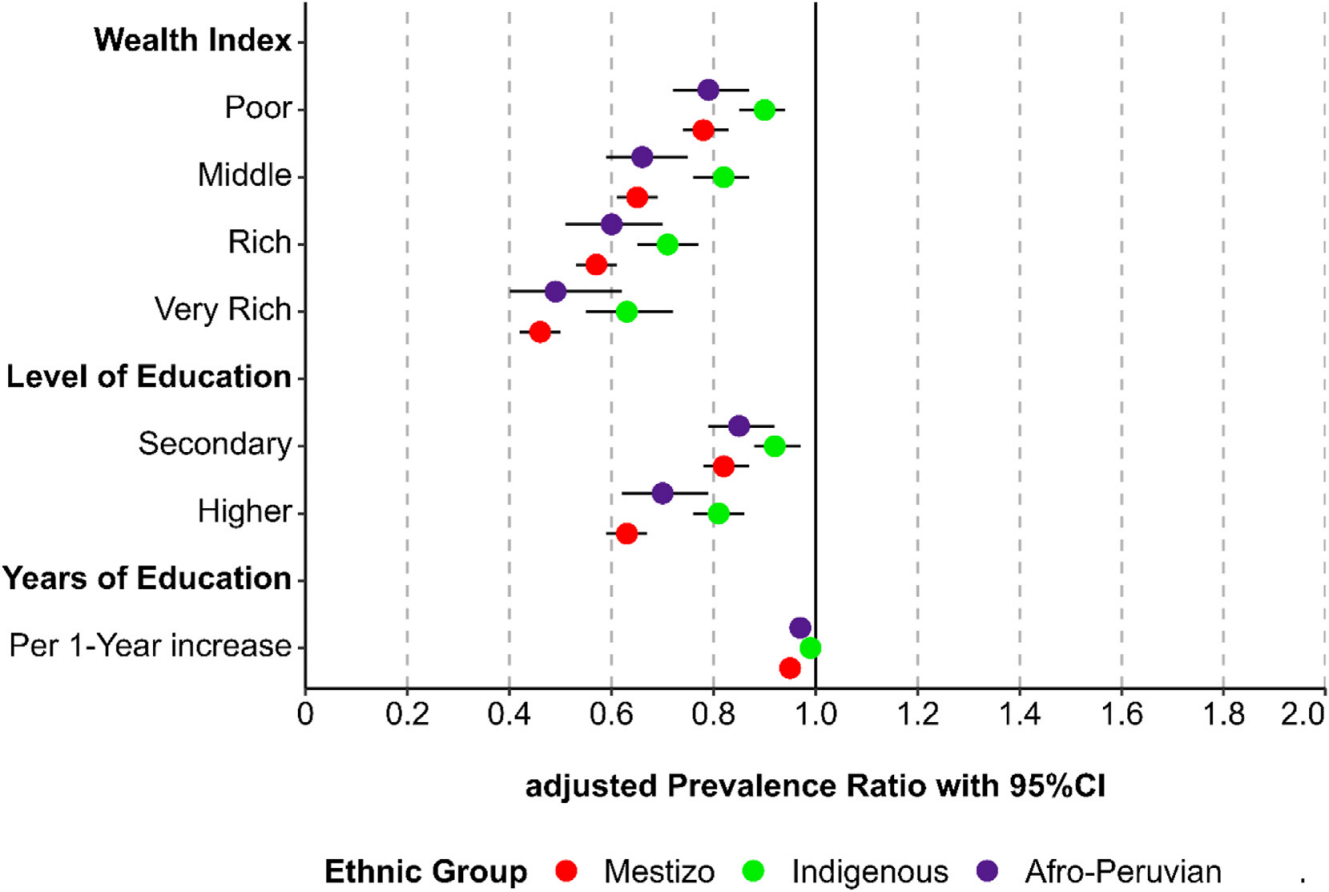
Association between maternal socioeconomic status and childhood anemia in Mestizo, Afro-Peruvian, and Indigenous individuals, 2017–2023 DHSs. **Note:** 95% CI: 95% confidence interval. Generalized linear model of Poisson family adjusted for child's age, mother's age, sex, area of residence, region of residence, family size, disability, partner status, employment status, and health insurance, stratified by ethnicity. Indigenous included Quechua, Aimara, and natives of the Amazon.

**Fig. 2:**
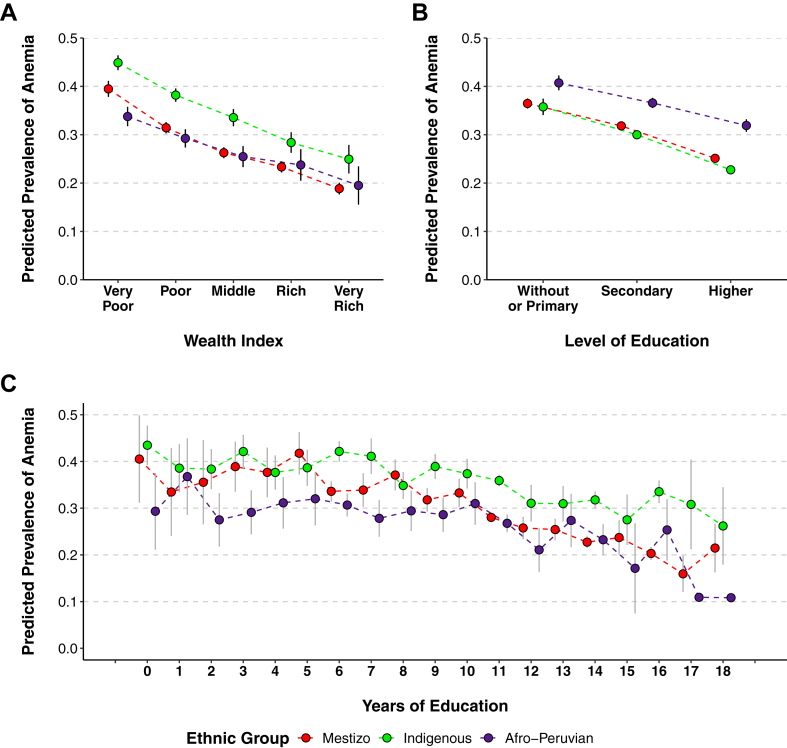
Predicted prevalence of childhood anemia according to socioeconomic status in Mestizo, Afro-Peruvian, and Indigenous individuals: (A) Wealth index, (B) Level of education, (C) Years of education, 2017–2023 DHSs. **Note:** Predicted prevalence estimates are based on generalized linear models of Poisson family were adjusted for child's age, mother's age, sex, area of residence, region of residence, family size, disability, partner status, employment status, and health insurance, stratified by ethnicity.

Table 2 and Fig. 1 display the adjusted estimates of the association between household wealth index and the prevalence of anemia among Peruvian children. Fig. 2A visualizes the predicted anemia prevalence based on wealth index, while Suppl. Table S3 presents the PRs across the Indigenous groups.

**Table 2:** Association between socioeconomic status and childhood anemia in Mestizo, Afro-Peruvian, and Indigenous groups, 2017–2023 DHSs.

Characteristics	Mestizo	Afro-Peruvian	Indigenous^a^
	PR (95% CI)	PR (95% CI)	PR (95% CI)
**Wealth index**			
Very poor	**Ref.**	**Ref.**	**Ref.**
Poor	0.78 (0.74–0.93)	0.79 (0.71–0.87)	0.90 (0.85–0.94)
Middle	0.65 (0.61–0.69)	0.66 (0.58–0.75)	0.82 (0.77–0.87)
Rich	0.57 (0.53–0.61)	0.60 (0.51–0.70)	0.71 (0.65–0.77)
Very rich	0.46 (0.42–0.50)	0.49 (0.40–0.62)	0.63 (0.56–0.72)
**Level of education**			
None or primary	**Ref.**	**Ref.**	**Ref.**
Secondary	0.82 (0.78–0.87)	0.85 (0.79–0.92)	0.93 (0.88–0.97)
Higher	0.63 (0.59–0.67)	0.70 (0.62–0.79)	0.81 (0.77–0.86)
**Years of education (per 1-year increase)**	0.95 (0.94–0.96)	0.97 (0.96–0.98)	0.98 (0.98–0.99)

PR: prevalence ratio. 95% CI: 95% confidence interval.

**Note:** Generalized linear model of Poisson family adjusted for child's age, mother's age, sex, area of residence, region of residence, family size, disability, partner status, employment status, and health insurance.

a Indigenous included Quechua, Aimara, and Native of the Amazon.

In Mestizo children, increasing household wealth was strongly associated with a reduced prevalence of anemia. Anemia dropped by 22% in poor households (PR = 0.78, 95% CI: 0.74–0.93) and 35% in middle-income households (PR = 0.65, 95% CI: 0.61–0.69) compared to those in very poor conditions. This association was stronger in rich and very rich households, reducing anemia by 43% (PR = 0.57, 95% CI: 0.53–0.61) and 54% (PR = 0.46, 95% CI: 0.42–0.50), respectively.

In Afro-Peruvian children, higher household wealth was associated with lower anemia prevalence, although the association was marginally weaker than in Mestizos. The prevalence of anemia was 21% lower in poor households (PR = 0.79, 95% CI: 0.71–0.87) and 34% lower in middle households (PR = 0.66, 95% CI: 0.58–0.75) compared to very poor households. Reductions reached 40% in rich (PR = 0.60, 95% CI: 0.51–0.70) and 51% in very rich households (PR = 0.49, 95% CI: 0.40–0.62).

For children of Indigenous mothers, the magnitude of the protective association between wealth index and anemia was substantially weaker. Children living in poor households had a 10% lower prevalence (PR = 0.90, 95% CI: 0.85–0.94), and those in middle households had an 18% reduction (PR = 0.82, 95% CI: 0.77–0.87), both compared to children from very poor households. Moreover, the prevalence of anemia decreased by 29% (PR = 0.71, 95% CI: 0.65–0.77) for children from rich households and by 37% (PR = 0.63, 95% CI: 0.56–0.72) for those from very rich households.

### Association between maternal education (level and years) with childhood anemia

Table 2 and Fig. 1 present the association between maternal education (level and years) and anemia in Peruvian children. Fig. 2B and C illustrate the predicted prevalence of anemia according to education variables, while Suppl. Table S3 provides the estimates across different Indigenous groups.

The protective association was robust in Mestizo children, in whom secondary and higher maternal education reduced the prevalence of anemia by 19% (PR = 0.81, 95% CI: 0.77–0.86) and 37% (PR = 0.63, 95% CI: 0.59–0.67), respectively. In contrast, Afro-Peruvian children whose mothers had completed secondary education had a 15% lower prevalence of anemia (PR = 0.85, 95% CI: 0.79–0.92) compared to children of mothers with only primary or no education. The association was stronger among children of mothers with higher education, who experienced a 30% lower prevalence of anemia (PR = 0.70, 95% CI: 0.62–0.79). Similarly, among children of Indigenous mothers, having maternal secondary education was weakly associated with a 7% lower prevalence of anemia (PR = 0.93, 95% CI: 0.88–0.97), while maternal higher education was linked to an 18% reduction in the prevalence of anemia (PR = 0.82, 95% CI: 0.78–0.87).

Concerning years of education, each additional year of maternal education was associated with a reduction of 5% the prevalence of anemia among Mestizo (PR = 0.95, 95% CI: 0.94–0.96), 3% in Afro-Peruvian (PR = 0.97, 95% CI: 0.96–0.98), and 2% in Indigenous children (PR = 0.98, 95% CI: 0.98–0.99). When separating Indigenous groups, the point prevalence reduction was consistent across the groups.

### Interaction analysis

Table 3 shows the interaction between SES proxies and ethnicity, indicating that the protective association of higher SES proxies and anemia is weaker for Afro-Peruvian and Indigenous children compared to Mestizos. Suppl. Table S4 presents the interactions of SES with different Indigenous groups in comparison to Mestizos.

**Table 3:** Interaction between maternal socioeconomic status and ethnicity (Afro-Peruvian and Indigenous vs. Mestizo) on childhood anemia in Peru, 2017–2023 DHSs.

	Ethnicity	PR (95% CI)	RERI (95% CI)
**Wealth index**			
Poor vs. very poor	Afro-Peruvian	1.08 (0.98–1.19)	0.42 (0.25–0.60)
	Indigenous^a^	1.09 (0.99–1.14)	0.14 (−0.01–0.28)
Middle vs. very poor	Afro-Peruvian	1.12 (1.00–1.26)	0.59 (0.40–0.78)
	Indigenous^a^	1.13 (1.04–1.22)	0.32 (0.17–0.48)
Rich vs. very poor	Afro-Peruvian	1.17 (1.00–1.37)	0.72 (0.49–0.95)
	Indigenous^a^	1.07 (0.97–1.18)	0.34 (0.17–0.50)
Very rich vs. very poor	Afro-Peruvian	1.19 (0.96–1.49)	0.85 (0.55–1.15)
	Indigenous^a^	1.17 (1.01–1.35)	0.55 (0.33–0.75)
**Level of education**			
Secondary vs. none or primary	Afro-Peruvian	1.08 (0.99–1.19)	0.39 (0.21–0.57)
	Indigenous^a^	1.08 (1.01–1.15)	0.71 (0.49–0.92)
Higher vs. none or primary	Afro-Peruvian	1.20 (1.06–1.36)	0.10 (−0.07–0.27)
	Indigenous^a^	1.23 (1.14–1.33)	0.46 (0.28–0.64)
**Years of education (per 1-year increase)**	Afro-Peruvian	1.028 (1.017–1.039)	0.033 (0.023–0.043)
	Indigenous^a^	1.030 (1.023–1.037)	0.030 (0.022–0.037)

PR: prevalence ratio; 95% CI: 95% confidence interval; RERI: relative excess prevalence due to interaction.

**Note:** Generalized linear model of Poisson family with an interaction term (SES × ethnicity) adjusted for child's age, mother's age, sex, area of residence, region of residence, family size, disability, partner status, employment status, and health insurance. Mestizos are the reference group for all comparisons. RERI measures whether the combined effect of SES and ethnicity on anemia is greater than (RERI > 0), equal to (RERI = 0), or less than (RERI < 0) the sum of their individual effects, which reflects an additive interaction.

a Indigenous includes Quechua, Aimara, and Native of the Amazon.

Among Afro-Peruvian children, PRs relative to Mestizos increased with household wealth, rising from 1.08 (95% CI: 0.98–1.19) in poor households to 1.19 (95% CI: 0.96–1.49) in very rich households, both compared to very poor households. Consistently, Indigenous children exhibited a comparable pattern, with PRs of 1.09 (95% CI: 0.99–1.14) in poor households and 1.17 (95% CI: 1.01–1.35) in very rich households. These estimates suggest that at the same wealth level, Afro-Peruvian and Indigenous children experience a persistently higher prevalence of anemia compared to Mestizos. The positive RERI values (range 0.14–0.85) further suggest a weaker protective association of wealth in these groups.

A similar trend is observed for maternal education. Among Afro-Peruvian children, having a mother with secondary education (vs. none or primary) is associated with an 8% higher prevalence of anemia (PR = 1.08, 95% CI: 0.99–1.19) relative to Mestizos, while higher maternal education is associated to a 20% increase (PR = 1.20, 95% CI: 1.06–1.36). In parallel, among Indigenous children, these associations are slightly stronger, with PRs of 1.08 (95% CI: 1.01–1.15) for secondary and 1.24 (95% CI: 1.15–1.34) for higher education. These findings suggest that even at higher education levels, Afro-Peruvian and Indigenous children continue to experience a greater prevalence of anemia than Mestizos. The RERI values (range 0.11–0.71) support this notion.

Each additional year of maternal education is associated with a small but significant increase in the prevalence of anemia among Afro-Peruvian (PR = 1.028, 95% CI: 1.017–1.039) and Indigenous children (PR = 1.030, 95% CI: 1.023–1.037) compared to Mestizos. The RERI values of 0.03 confirm that, relative to Mestizos, Afro-Peruvian, and Indigenous children gain less protection from increased maternal education.

### Annual variations in prevalence ratios, predicted prevalences, and interaction

The annual estimation of PR exhibited a stagnant trend, maintaining a consistent direction and magnitude from 2017 to 2023, with a stronger association among Mestizos, as illustrated in Suppl. Figure S3. Consequently, the predicted prevalence of childhood anemia showed little change over these years, remaining substantially higher among Indigenous children (Suppl. Figure S4). Regarding interaction analysis, although Afro-Peruvian and Indigenous individuals had a higher prevalence of anemia than Mestizo individuals at the same wealth index, this difference was not significant in most years due to the wide 95% CI. However, at the same level and years of education, these two groups exhibited a significantly higher prevalence compared to Mestizo individuals (Suppl. Figure S5).

## Discussion

Our study confirms that higher maternal SES, as indicated by education or wealth index, is associated with a lower prevalence of anemia in Peruvian children, but the magnitude of this association is significantly weaker for Afro-Peruvian and Indigenous populations compared to Mestizos. Wealthy and educated mothers, yet anemic children? This is the reality for many Afro-Peruvian and Indigenous peoples. This inequity is concerning in Peru, given its large ethnoracial heterogeneity. Inadvertently, increasing SES alone will not erase ethnic disparities, but rather may worsen them. In explicit terms, improvements in SES are associated with greater reductions in anemia among Mestizos, ultimately deepening disparities. These findings confirm the occurrence of the *marginalization-related diminished returns* phenomenon in Peru. Here, we first explore the plausibility of the protective role of SES and then examine the *differential exposures* and *differential effects* experienced by Afro-Peruvian and Indigenous individuals.

Maternal SES is associated with both direct protective effect on children's health outcomes and indirect effects through improved access to resources, better living conditions, and the promotion of health-supporting behaviors across lifespan.^32^, ^33^, ^34^ Conversely, people with low SES are more susceptible to unfavorable health outcomes. In this study, we found that a higher SES was associated with a lower prevalence of childhood anemia. This could be attributable to the higher intake of protein and iron from animal sources among children of more educated mothers, who are also more likely to have access to updated nutritional information.^35^ Likewise, children living in rich households are exposed to more micronutrient intake, healthier food consumption, better food preparation facilities, and more nutritional knowledge.^36^ Educated mothers tend to live in affluent households, typically located in urban areas with access to clean water, sanitation, healthcare, economic opportunities, reliable transportation, modern infrastructure, social services, and greater exposure to information and technology.^37^ However, the extent to which SES translates into these benefits varies by ethnicity, as differences in exposures and effects limit its impact on marginalized ethnic groups.

Mothers self-identifying as Afro-Peruvian and Indigenous are disproportionately exposed to adverse social determinants of health. One major factor contributing to these disparities is unequal access to education, which is linked to disparities in school quality, learning environments, and opportunities for higher education. Despite the advances in education, female children in rural areas often begin assisting their mothers from a young age, leading to frequent delays or interruptions in their schooling. In many households, educational resources are prioritized for male children, further restricting female children's future access to formal employment.^38^ Enduring gender inequities continues to result in delayed or interrupted education for girls, perpetuating cycles of poverty and limited professional opportunities.^39^^,^^40^ Additionally, childhood health conditions that impair concentration or cognitive function can limit educational gains.^4^ Many of these mothers began their education nearly two decades ago, when illiteracy rates were very high and anemia affected over 60% of children in rural areas.^39^^,^^41^

One year of education for Afro-Peruvian and Indigenous women is not comparable to one year of education for Mestizo women. Commonly used measures of education focus on years of schooling but fail to account for differences in school quality and learning outcomes. The key question is: to what extent does schooling translate into meaningful education? Students from different ethnic backgrounds who have spent the same number of years in school often achieve vastly different learning outcomes. This distinction between schooling (formal years spent in school) and education (knowledge and skills acquired) is critical.^42^ Simply counting years of schooling without considering these disparities is insufficient for meaningful comparisons of education attainment across populations.^43^^,^^44^ School quality is particularly low in disadvantaged ethnic communities due to limited resources, short-term teacher contracts and inadequate teacher training.^45^ These challenges are further exacerbated by rurality and remoteness, where approximately four out of ten Afro-Peruvian and Indigenous mothers reside.

Economic disparities also play a critical role in shaping childhood anemia. The wealth index—encompassing wealth accumulation, quality of assets, and stability—is unevenly distributed among ethnic groups, limiting the protective effect of SES. Historically disadvantaged ethnic groups may be limited in how they can deploy their wealth to attain access to resources, even if they have similar wealth levels (i.e., wealth does not afford the same opportunities). Even when Indigenous families fall within the same wealth quintile as Mestizo families, their assets may be of lower quality, less stable, or provide fewer long-term benefits. For Afro-Peruvian, interestingly, the difference in the protective effect of wealth index, compared to Mestizos, was found to be negligible. Some features may be overlooked by the wealth index, which fails to capture asset quality and its tangible benefits.

Over the past five decades, large-scale migration from rural to urban areas has required cultural assimilation. Many Indigenous migrants have learned Spanish, but language acquisition alone does not ensure full integration. Linguistic barriers, the preservation of Indigenous health beliefs, and the government's failure to integrate traditional and Western medicine has deepened mistrust, reinforcing divisions and complicating integration.^46^^,^^47^ This mistrust is fueled by social and cultural divides that separate this population from healthcare providers and services. Predictably, non-Spanish speakers are more likely to not know their human health rights.^48^ As a result, many individuals from these communities hesitate to seek healthcare services or participate in government programs designed to improve their well-being. Taken together, these factors directly impact access to essential resources, including healthy food, necessary for maintaining proper nutrition.^49^ The Aguarunas' diet, for example, is mostly based on cassava and bananas, with scarce animal protein.^50^ Hence, these families are more prone to food insecurity and, in turn, anemia.^51^

Discrimination compounds these challenges, with many Afro-Peruvian and Indigenous individuals facing exclusion in healthcare and workplace settings. Despite primarily residing in urban areas, 70% of Afro-Peruvians who fell ill in the past year did not seek medical care due to limited access and concerns about discrimination.^52^ Likewise, Indigenous individuals, who are based mostly in rural areas, were more than twice as likely to experience maltreatment in healthcare facilities, further deepening their mistrust in the system.^17^ This extends to the labor market, where negative stereotypes and biases against Indigenous individuals lead to exclusionary hiring practices, in which their professional abilities are underestimated, limiting their access to well-paid, high-skilled jobs.^53^ Most non-Spanish speakers are informally employed in agriculture, livestock, forestry, retail trade, and domestic service in private households—sectors that are among the lowest-paying.^25^

In addition to structural and social discrimination, ongoing internal violence, such as labor exploitation and illegal mining, further worsens conditions for Indigenous communities.^54^ Women and girls, in particular, are vulnerable to exploitation in agricultural and domestic work, where they face physical or verbal abuse, threats, and other forms of maltreatment that harm their health and well-being.^55^ Illegal mining, by contrast, leads to food insecurity, water contamination, displacement of communities, and exposure to toxic pollutants. For instance, Harákmbut children—members of an Amazonian Indigenous community—are directly affected by illegal mining and have been found to have high mercury levels, anemia and micronutrient deficiencies.^56^

The fragmentation and segmentation of the Peruvian healthcare system also lead to diminished returns.^57^ Indigenous communities often rely on nearby facilities that provide only basic care, while staff shortages, language barriers, and cultural differences create conflicts between medical personnel and patients.^58^ Only one in ten newly graduated doctors have a basic understanding of native languages like Quechua or Aimara, hindering communication.^59^^,^^60^ Furthermore, in remote regions such as Loreto, Puno, and Cusco—where a large proportion of Indigenous population resides—healthcare staffing remains inadequate, with only 18–26 personnel per 10,000 inhabitants, far below the WHO recommendations.^45^^,^^61^ This shortage is especially noticeable in rural primary care facilities, 92% of which lack a nutritionist, further hindering the implementation of anemia prevention programs in these communities.^62^ Beyond personnel limitations, half of the Indigenous communities live over an hour away from the nearest health facility.^16^ All in all, these overlapping factors—marginalization, economic exclusion, discrimination, and violence—create a cycle of disadvantage that limits opportunities and their effects on Indigenous and Afro-Peruvian populations, which is further exacerbated by the weaknesses in the healthcare system.

For a judicious interpretation of this study, some limitations should be acknowledged. First, its cross-sectional design does not allow the establishment of a causal relationship between maternal SES and childhood anemia, limiting our findings to associations observed at the time of data collection. However, this design enables obtaining response to our research question in a representative sample of the Peruvian population.^63^ Given the logical causal structure and the nature of our variables, this study offers valuable insights into the causal relationships examined, despite its cross-sectional design. Second, the exposure variables—education and wealth index—are proxies of SES and may not fully capture the complexities of economic stability, income variability, or access to liquid assets that directly influence SES.^28^ Third, although the study includes maternal education as a SES indicator, it does not account for the quality of education received. A more comprehensive measure is Learning-Adjusted Years of Schooling, which captures both the quantity and quality of education and should be examined in future studies.^43^ Educational quality is a critical factor that can influence not only maternal skills and knowledge in managing and preventing child anemia but also future employment opportunities and income levels. In many contexts, the reputation and accreditation of educational institutions, as well as the relevance of their curricula, can significantly affect access to stable, well-paid jobs, which indirectly impacts child health.^64^ Fourth, although all these analyses were adjusted for the same covariates, due to the underrepresentation of some Indigenous groups, these analyses were exploratory in nature. Despite these limitations, our study analyzed a representative sample of mothers from various ethnic and socioeconomic backgrounds, providing a valuable approximation based on Peruvian data and offering essential information for the development of child health policies aimed at marginalized groups. Finally, we used three indirect measures of SES, obtaining similar results.

### Conclusion

Higher maternal SES was associated with a lower prevalence of childhood anemia in Peru. This association was measured with maternal level of education, years of education, and wealth index, with all three being consistent; however, they were found to be inadvertently weaker for Afro-Peruvian and Indigenous compared to Mestizo groups. Thus, increasing SES alone will not eradicate ethnic disparities and may, in fact, exacerbate them. This aligns with the marginalization-related diminished returns theory. Hence, it is imperative to recognize the *differential exposures* and *differential effects* that Afro-Peruvian and Indigenous individuals are subject to and address them through policies at economic, public, and social levels. This will allow these communities to mobilize their SES to ensure better health outcomes. Otherwise, systemic barriers will persist, reinforcing historical grievances and contributing to social fragmentation.

## Contributors

AAC: Conceptualization, methodology, investigation, visualization, data curation, formal analysis, and writing-original draft. CIE: Conceptualization, methodology, data curation, visualization, formal analysis, writing-original draft. PRV: Investigation, writing-original draft. DUP: Conceptualization, investigation, writing-original draft. BC: Investigation, supervision, validation, and writing–review & editing. AAC and CIE accessed and verified the underlying data. AAC was responsible for the decision to submit the manuscript.

## Data sharing statement

The database of the Peruvian DHS is publicly available in Spanish language at https://proyectos.inei.gob.pe/microdatos/.

## Declaration of interests

The authors declare no conflicts of interest.

## Appendix A Supplementary data

Abstract in Spanish

Suppl. Figures and Suppl. Tables
