# Supplementary material for: Ethnic disparities in the association between maternal socioeconomic status and childhood anemia in Peru: a nationwide multiyear cross-sectional study
Source: Lancet Reg Health Am. 2025 May 23;47:101117. doi: 10.1016/j.lana.2025.101117 (PMC12276629; doi:10.1016/j.lana.2025.101117)
Supplement: Abstract in Spanish [file mmc1.docx]

**Editorial disclaimer**

The translation of the Summary was submitted by the authors, and we reproduce it as supplied. It has not been peer reviewed. Our editorial processes have only been applied to the original version in English, which should serve as a reference for this manuscript.

**RESUMEN**

**Antecedentes:** El nivel socioeconómico (NSE) materno está estrechamente vinculado con los desenlaces de salud infantil. Sin embargo, la teoría de los retornos disminuidos por marginación sugiere que los incrementos en el NSE generan menores beneficios en salud para poblaciones marginadas, tales como los grupos afroperuanos e indígenas, en comparación con grupos mayoritarios como los mestizos, en gran parte debido a barreras sistémicas y desventajas sociales. Por lo tanto, el presente estudio tuvo como objetivo explorar las disparidades étnicas en la asociación entre el NSE materno y la anemia infantil en el Perú.

**Métodos:** Se utilizó información de la Encuesta Demográfica y de Salud Familiar del Perú (ENDES) del 2017 al 2023 para realizar un estudio transversal analítico que incluyó a niños de 6 a 59 meses y sus respectivas madres. La variable etnicidad se agrupó en mestizo, afroperuano e indígena (quechua, aimara y nativo amazónico). Se emplearon tres indicadores del NSE: índice de riqueza, nivel educativo y años de educación. Tras estratificar por grupo étnico, se estimaron razones de prevalencia (RP) ajustadas con sus respectivos intervalos de confianza al 95% (IC 95%) mediante modelos lineales generalizados con familia *Poisson*. La interacción se evaluó en escalas multiplicativa y aditiva.

**Resultados:** De un total de 234,364 madres peruanas, el 45.7% (n = 107,118) se identificaron como mestizas, el 12.6% (n = 29,557) como afroperuanas y el 41.7% (n = 97,689) como indígenas. La prevalencia global de anemia infantil fue de 32.2%. La asociación entre un índice de riqueza muy alto y una menor prevalencia de anemia fue más débil entre los indígenas (RP = 0.63, IC 95%: 0.56–0.72) en comparación con los mestizos (RP = 0.46, IC 95%: 0.42–0.50). De forma similar, la asociación entre mayor nivel educativo materno y menor prevalencia de anemia fue menos marcada en afroperuanos (RP = 0.70, IC 95%: 0.62–0.79) e indígenas (RP = 0.81, IC 95%: 0.77–0.86) que en mestizos (RP = 0.63, IC 95%: 0.59–0.67). Se observó un patrón similar respecto a los años de educación materna (mestizos [RP = 0.95, IC 95%: 0.94–0.96], afroperuanos [RP = 0.97, IC 95%: 0.96–0.98], e indígenas [RP = 0.98, IC 95%: 0.98–0.99]). El análisis de interacción confirmó asociaciones significativamente más débiles para afroperuanos e indígenas en comparación con los mestizos.

**Interpretación:** El NSE materno se asocia con menor prevalencia de anemia infantil, siendo de mayor magnitud entre la población mestiza en comparación con los grupos afroperuano e indígena. Este patrón es consistente con la teoría de los retornos disminuidos por marginación. Elevar el NSE por sí solo no eliminaría las desigualdades étnicas, e incluso podría ampliarlas, lo que resalta la necesidad de intervenciones centradas en la equidad que aborden las barreras estructurales y sistémicas subyacentes.

**Financiamiento:** Autofinanciado.

**Palabras clave:** Indígenas; Mestizos; Afroperuanos; Nivel Socioeconómico; Grupos Étnicos; Nivel Educativo; Desigualdades; Perú.
