# Supplementary material for: Ethnic disparities in the association between maternal socioeconomic status and childhood anemia in Peru: a nationwide multiyear cross-sectional study
Source: Lancet Reg Health Am. 2025 May 23;47:101117. doi: 10.1016/j.lana.2025.101117 (PMC12276629; doi:10.1016/j.lana.2025.101117)
Supplement: Suppl. Figures and Suppl. Tables [file mmc2.pdf]

Ethnic Disparities in the Association Between Maternal Socioeconomic Status and Childhood Anemia in Peru: A Nationwide Multiyear Cross-Sectional Study

Table of Contents

Supplementary Figure 1. Direct Acyclic Graph (DAG)…………………………………………….…2

Supplementary Figure 2. Participant selection flowchart, 2017–2023 DHSs……………………………3

Supplementary Figure 3. Annual variation in the prevalence ratio of maternal socioeconomic status on childhood anemia in Mestizo, Afro-Peruvian and Indigenous individuals: (A) Wealth index, (B) Level of education, (C) Years of education, 2017–2023 DHSs……………………………………………………4

Supplementary Figure 4. Annual variation in the predicted prevalence of childhood anemia according to socioeconomic status in Mestizo, Afro-Peruvian and Indigenous individuals: (A) Wealth index, (B) Level of education, 2017–2023 DHSs……………………………………………………………………….5

Supplementary Figure 5. Annual variation of interaction between ethnicity and maternal socioeconomic status on childhood anemia (Afro-Peruvian and Indigenous, versus Mestizo individuals), 2017–2023 DHSs…………………………………………………………………………………………………..6

Supplementary Table 1. STROBE Statement…………………………………………………………….7

Supplementary Table 2. Characteristics of Indigenous groups (Quechua, Aimara, and Native of the Amazon individuals), 2017–2023 DHSs……………………………………...………………………9

Supplementary Table 3. Association between maternal socioeconomic status and childhood anemia in Indigenous groups (Quechua, Aimara, and Native of the Amazon vs. Mestizo), 2017–2023 DHSs……..10

Supplementary Table 4. Interaction between maternal socioeconomic status and ethnicity (Quechua, Aimara, and Native of the Amazon vs. Mestizo) on childhood anemia in Peru, 2017–2023 DHSs………11

Supplementary Figure 1. Direct Acyclic Graph (DAG)

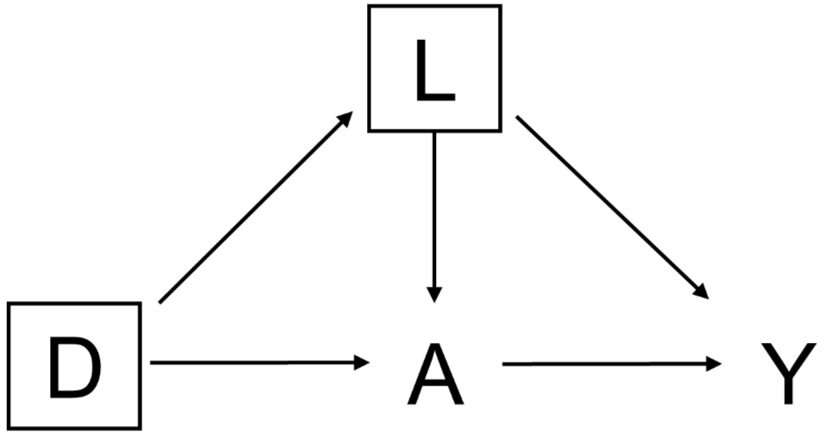

A: Exposure (socioeconomic status); Y: outcome (childhood anemia), L: confounders (child's age, mother's age, sex, area of residence, region of residence, family size, disability, partner status, employment status, and health insurance); D: ethnicity.
Note: D was conditioned on by design, while L was conditioned on during the analysis.

Supplementary Figure 2. Participant selection flowchart, 2017–2023 DHSs

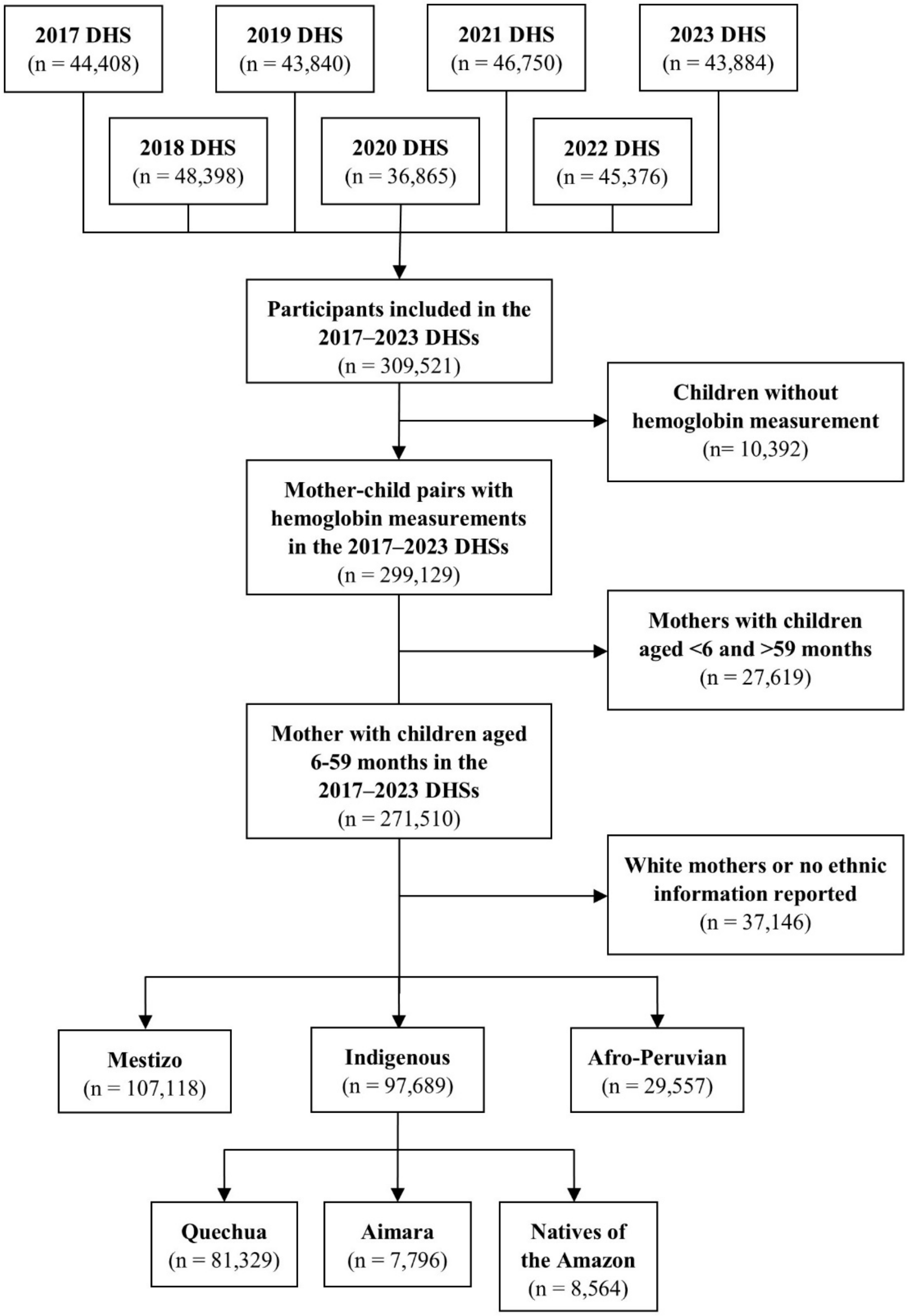

Supplementary Figure 3. Annual variation in the prevalence ratio of maternal socioeconomic status on childhood anemia in Mestizo, Afro-Peruvian and Indigenous individuals: (A) Wealth index, (B) Level of education, (C) Years of education, 2017–2023 DHSs

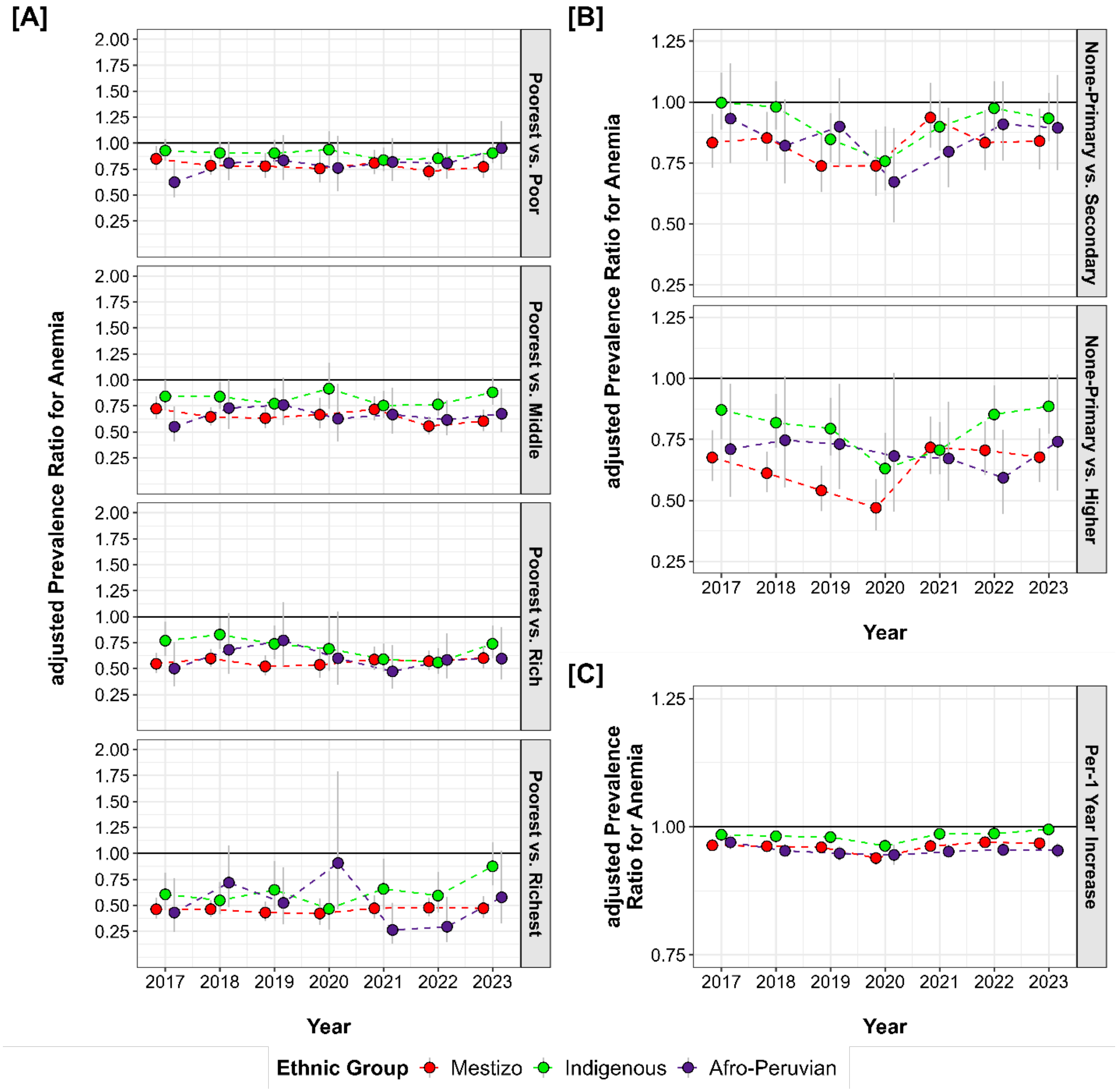

Note: Generalized linear models of Poisson family were adjusted for child's age, mother's age, sex, area of residence, region of residence, family size, disability, partner status, employment status, and health insurance, stratified by ethnicity.

Supplementary Figure 4. Annual variation in the predicted prevalence of childhood anemia according to socioeconomic status in Mestizo, Afro-Peruvian and Indigenous individuals: (A) Wealth index, (B) Level of education, 2017–2023 DHSs

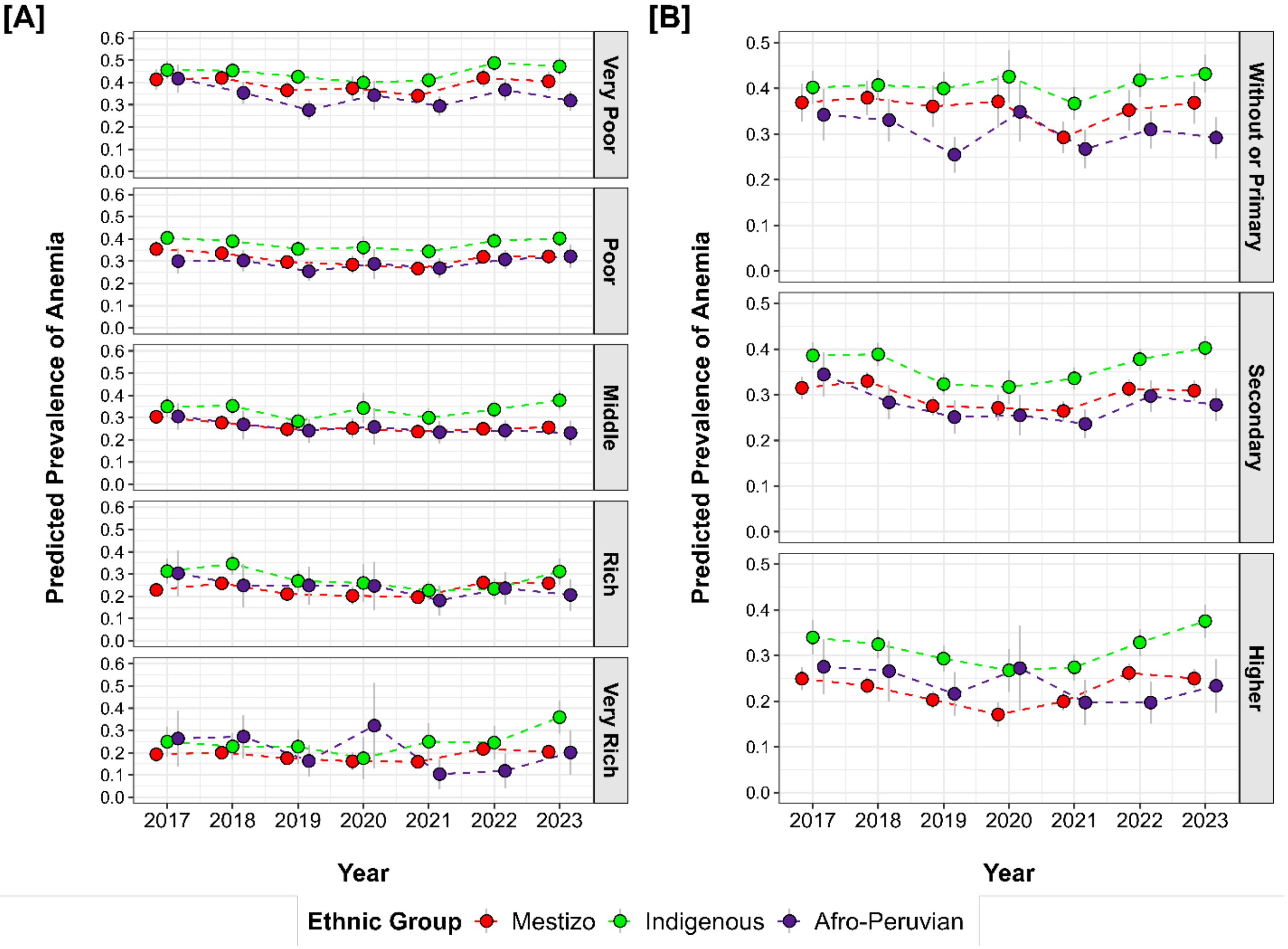

Note: Predicted prevalence estimates are based on generalized linear models of Poisson family were adjusted for child's age, mother's age, sex, area of residence, region of residence, family size, disability, partner status, employment status, and health insurance, stratified by ethnicity.

Supplementary Figure 5. Annual variation of interaction between ethnicity and maternal socioeconomic status on childhood anemia (Afro-Peruvian and Indigenous, versus Mestizo individuals), 2017–2023 DHSs

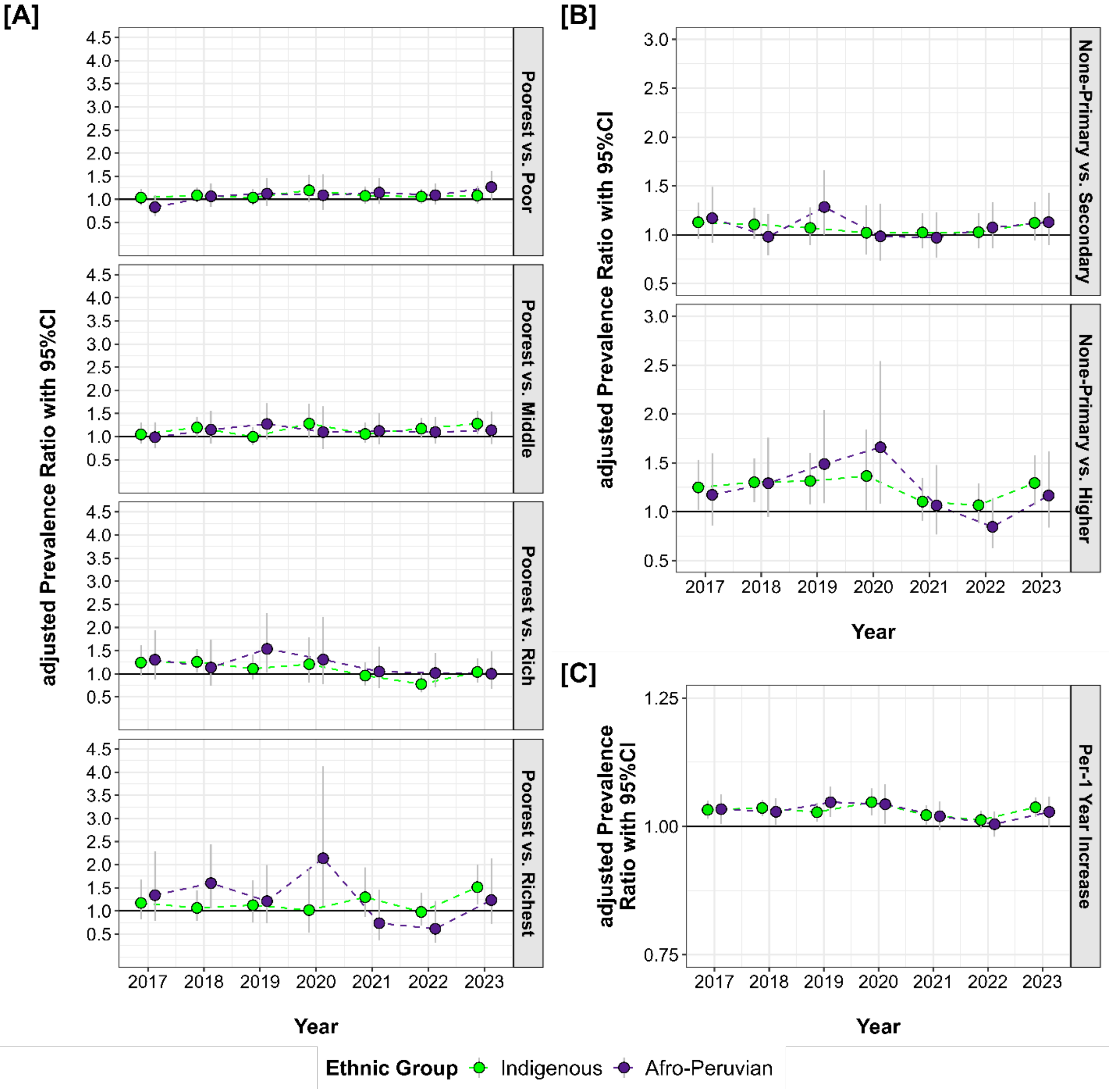

Note: Generalized linear model of Poisson family with an interaction term (SES x ethnicity) adjusted for child's age, mother's age, sex, area of residence, region of residence, family size, disability, partner status, employment status, and health insurance. Mestizos were the reference for all comparisons.

Supplementary Table 1. STROBE Statement

	Item No	Recommendation	Section
**Title and abstract**	1	(*a*) Indicate the study's design with a commonly used term in the title or the abstract	Title page
		(*b*) Provide in the abstract an informative and balanced summary of what was done and what was found	Abstract
**Introduction**			
Background/rationale	2	Explain the scientific background and rationale for the investigation being reported	Introduction
Objectives	3	State specific objectives, including any prespecified hypotheses	Introduction
**Methods**			
Study design	4	Present key elements of study design early in the paper	Methods
Setting	5	Describe the setting, locations, and relevant dates, including periods of recruitment, exposure, follow-up, and data collection	Methods
Participants	6	(*a*) Give the eligibility criteria, and the sources and methods of selection of participants	Methods
Variables	7	Clearly define all outcomes, exposures, predictors, potential confounders, and effect modifiers. Give diagnostic criteria, if applicable	Methods
Data sources/ measurement	8*	For each variable of interest, give sources of data and details of methods of assessment (measurement). Describe comparability of assessment methods if there is more than one group	Methods
Bias	9	Describe any efforts to address potential sources of bias	Methods
Study size	10	Explain how the study size was arrived at	Methods
Quantitative variables	11	Explain how quantitative variables were handled in the analyses. If applicable, describe which groupings were chosen and why	Methods
Statistical methods	12	(*a*) Describe all statistical methods, including those used to control for confounding	Methods
		(*b*) Describe any methods used to examine subgroups and interactions	Methods
		(*c*) Explain how missing data were addressed	Methods
		(*d*) If applicable, describe analytical methods taking account of sampling strategy	Methods
		(*e*) Describe any sensitivity analyses	Methods
**Results**			
Participants	13*	(a) Report numbers of individuals at each stage of study—eg numbers potentially eligible, examined for eligibility, confirmed eligible, included in the study, completing follow-up, and analysed	Results
		(b) Give reasons for non-participation at each stage	Suppl. Material
		(c) Consider use of a flow diagram	Suppl. Material
Descriptive data	14*	(a) Give characteristics of study participants (eg demographic, clinical, social) and information on exposures and potential confounders	Results
		(b) Indicate number of participants with missing data for each variable of interest	Results
Outcome data	15*	Report numbers of outcome events or summary measures	Results
Main results	16	(*a*) Give unadjusted estimates and, if applicable, confounder-adjusted estimates and their precision (eg, 95% confidence interval). Make clear which confounders were adjusted for and why they were included	Results
		(*b*) Report category boundaries when continuous variables were categorized	NA
		(*c*) If relevant, consider translating estimates of relative risk into absolute risk for a meaningful time period	NA

Other analyses	17	Report other analyses done—eg analyses of subgroups and interactions, and sensitivity analyses	Results
**Discussion**			
Key results	18	Summarise key results with reference to study objectives	Discussion
Limitations	19	Discuss limitations of the study, taking into account sources of potential bias or imprecision. Discuss both direction and magnitude of any potential bias	Discussion
Interpretation	20	Give a cautious overall interpretation of results considering objectives, limitations, multiplicity of analyses, results from similar studies, and other relevant evidence	Discussion
Generalisability	21	Discuss the generalisability (external validity) of the study results	Discussion
**Other information**			
Funding	22	Give the source of funding and the role of the funders for the present study and, if applicable, for the original study on which the present article is based	Methods

Note: An Explanation and Elaboration article discusses each checklist item and gives methodological background and published examples of transparent reporting. The STROBE checklist is best used in conjunction with this article (freely available on the Web sites of PLoS Medicine at http://www.plosmedicine.org/, Annals of Internal Medicine at http://www.annals.org/, and Epidemiology at http://www.epidem.com/). Information on the STROBE Initiative is available at www.strobe-statement.org.

Supplementary Table 2. Characteristics of Indigenous groups (Quechua, Aimara, and Native of the Amazon individuals), 2017–2023 DHSs

Characteristics	Quechua (n = 82,573)	Aimara (n = 7,957)	Natives of the Amazon (n = 8,665)
	n (%)	n (%)	n (%)
**Mother's age (mean [SD])**	33.70 [7.6]	32.93 [8.9]	32.53 [7.4]
**Level of education**			
None or primary	30,373 (33.0)	1,516 (20.9)	4,951 (57.6)
Secondary	36,132 (44.8)	4,616 (57.1)	3,086 (35.1)
Higher	16,068 (22.2)	1,825 (22.1)	628 (7.3)
**Wealth index**			
Poorest	37,554 (36.7)	1,393 (25.6)	7,275 (80.6)
Poor	22,724 (27.5)	2,656 (31.0)	934 (10.2)
Middle	12,457 (18.2)	2,337 (24.5)	251 (4.2)
Rich	6,607 (11.2)	1,141 (13.5)	130 (2.2)
Richest	3,231 (6.4)	430 (5.3)	75 (2.9)
**Area of residence**			
Urban	43,384 (60.7)	6,058 (67.2)	1,899 (24.2)
Rural	39,189 (39.3)	1,899 (32.8)	6,766 (75.8)
**Region of residence**			
Lima	5,483 (19.8)	191 (9.5)	242 (8.9)
Resto of Coast	7,425 (8.8)	4,705 (28.4)	112 (1.5)
Highland	57,677 (60.6)	2,802 (59.8)	59 (0.9)
Jungle	11,988 (10.8)	259 (2.3)	8,252 (88.7)
**Physical or mental disability?**			
None	82,061 (99.4)	7,911 (99.5)	8,625 (99.4)
At least one	512 (0.6)	46 (0.5)	40 (0.6)
**Partner status**			
No	10,072 (12.3)	1,134 (14.1)	638 (7.2)
Yes	72,501 (87.7)	6,823 (85.9)	8,027 (92.8)
**Employment status**			
Not working	20,175 (26.1)	2,062 (23.2)	3,002 (31.8)
Working	6,447 (8.2)	645 (7.4)	384 (4.5)
Work in last year	52,837 (65.7)	4,992 (69.4)	5,165 (63.7)
**Family size (members)**			
≤4	31,579 (37.8)	3,941 (49.9)	1,664 (18.1)
>4	50,994 (62.2)	4,016 (50.1)	7,001 (81.9)
**Health Insurance**			
No	7,850 (11.9)	1,419 (19.2)	1,028 (13.8)
Yes	74,723 (88.1)	6,538 (80.8)	7,637 (86.2)
**Sex of Children**			
Male	42,090 (51.1)	4,128 (51.9)	4,434 (51.3)
Female	40,483 (48.9)	3,829 (48.1)	4,231 (48.7)
**Child's age (mean [SD])**	34.06 [15.5]	33.95 [15.3]	35.21 [15.4]
**Anemia**			
No	49,304 (62.1)	5,008 (56.2)	4,434 (51.3)
Yes	32,025 (37.9)	2,788 (43.8)	4,231 (48.7)

SD: standard deviation

Note: Proportions and means are weighted to account for the complex survey design.

Supplementary Table 3. Association between maternal socioeconomic status and childhood anemia in Indigenous groups (Quechua, Aimara, and Native of the Amazon vs. Mestizo), 2017–2023 DHSs

**Characteristics**	**Quechua**	**Aimara**	**Natives of the Amazon**
	**PR (95%CI)**	**PR (95%CI)**	**PR (95%CI)**
**Wealth index**			
Very poor	**Ref.**	**Ref.**	**Ref.**
Poor	0.93 (0.88–0.98)	0.82 (0.70–0.96)	0.72 (0.60–0.87)
Middle	0.83 (0.77–0.89)	0.78 (0.63–0.95)	1.03 (0.73–1.44)
Rich	0.71 (0.65–0.79)	0.78 (0.61–0.99)	0.61 (0.28–1.34)
Very rich	0.65 (0.56–0.74)	0.68 (0.48–0.96)	0.40 (0.18–0.87)
**Level of education**			
None or primary	**Ref.**	**Ref.**	**Ref.**
Secondary	0.91 (0.86–0.96)	1.13 (0.95–1.35)	1.02 (0.89–1.16)
Higher	0.79 (0.75–0.84)	1.02 (0.84–1.24)	0.97 (0.78–1.20)
**Years of education (per 1-year increase)**	0.98 (0.97–0.99)	1.01 (0.99–1.03)	0.99 (0.97–1.01)

PR: prevalence ratio. 95% CI: 95% confidence interval.

Note: Generalized linear model of Poisson family adjusted for child's age, mother's age, sex, area of residence, region of residence, family size, disability, partner status, employment status, and health insurance.

Supplementary Table 4. Interaction between maternal socioeconomic status and ethnicity (Quechua, Aimara, and Native of the Amazon vs. Mestizo) on childhood anemia in Peru, 2017–2023 DHSs

Characteristics	Ethnicity	PR (95%CI)	RERI (95%CI)
**Wealth index**			
Poor vs. Very poor	Quechua	1.12 (1.05–1.20)	0.24 (0.09 – 0.39)
	Aimara	0.94 (0.81–1.09)	-0.27 (-0.55 – 0.01)
	Native of the Amazon	0.79 (0.66–0.95)	-0.29 (-0.52 - -0.08)
Middle vs. Very poor	Quechua	1.17 (1.08–1.28)	0.42 (0.26 – 0.58)
	Aimara	0.99 (0.84–1.16)	-0.09 (-0.39 – 0.20)
	Native of the Amazon	1.09 (0.72–1.65)	0.12 (-0.37 – 0.60)
Rich vs. Very poor	Quechua	1.11 (1.00–1.23)	0.43 (0.26 – 0.60)
	Aimara	1.07 (0.86–1.34)	0.07 (-0.28 – 0.41)
	Native of the Amazon	0.77 (0.32–1.85)	-0.12 (-0.82 – 0.57)
Very rich vs. Very poor	Quechua	1.23 (1.05–1.43)	0.66 (0.43 – 0.89)
	Aimara	1.10 (0.79–1.53)	0.21 (-0.24 – 0.65)
	Native of the Amazon	0.60 (0.27–1.30)	-0.19 (-0.67 – 0.30)
**Level of education**			
Secondary Vs. None or primary	Quechua	1.07 (1.00–1.15)	0.13 (-0.05 – 0.30)
	Aimara	1.25 (1.05–1.49)	0.26 (-0.14 – 0.67)
	Native of the Amazon	1.09 (0.96–1.24)	-0.04 (-0.28 – 0.22)
Higher Vs. None or primary	Quechua	1.25 (1.15–1.35)	0.50 (0.32 – 0.69)
	Aimara	1.42 (1.16–1.73)	0.63 (0.18 – 1.09)
	Native of the Amazon	1.23 (1.01–1.50)	0.35 (0.01 – 0.69)
**Years of education (per 1-year increase)**	Quechua	1.03 (1.02–1.04)	0.03 (0.02 – 0.4)
	Aimara	1.04 (1.03–1.06)	0.04 (0.03 – 0.06)
	Native of the Amazon	1.02 (1.01–1.04)	0.02 (-0.01 – 0.04)

PR: prevalence ratio; 95% CI: 95% confidence interval; RERI: relative excess prevalence due to interaction.
Note: Generalized linear model of Poisson family with an interaction term (SES x ethnicity) adjusted for child's age, mother's age, sex, area of residence, region of residence, family size, disability, partner status, employment status, and health insurance. Mestizos were the reference for all comparisons. RERI measures whether the combined effect of SES and ethnicity on anemia is greater than (RERI > 0), equal to (RERI = 0), or less than (RERI < 0) the sum of their individual effects, which reflects an additive interaction.
